# Long noncoding RNA Gomafu upregulates Foxo1 expression to promote hepatic insulin resistance by sponging miR-139-5p

**DOI:** 10.1038/s41419-018-0321-7

**Published:** 2018-02-19

**Authors:** Caifeng Yan, Jin Li, Shangyong Feng, Ying Li, Lu Tan

**Affiliations:** 1 Department of Endocrinology, Clinical Medical College of Yangzhou University, Yangzhou, China; 2 Department of Enphrology, Clinical Medical College of Yangzhou University, Yangzhou, China

## Abstract

Long non-coding RNA Gomafu is involved in diabetes-related diseases. However, its role in insulin resistance (IR) remains unclear. Our objective is to explore the role of Gomafu in hepatic IR and glucose intolerance. Gomafu expression was determined in livers of ob/ob mice and high-fat diet (HFD) mice. The binding activity of NF-κB on the *Gomafu* promoter was measured by chromatin immunoprecipitation and quantitative real-time PCR assays. Increased Gomafu expression was observed in the livers of obese mice. Besides, the binding of NF-κB on the *Gomafu* promoter was also observed in hepatocytes from ob/ob mice. Further study showed that knockdown of NF-κB p65 alleviated the increase in hepatic Gomafu expression in vivo and in vitro. Knockdown of hepatic Gomafu inhibited hepatic glucose production (HGP) and improved insulin sensitivity in obese mice, whereas, overexpression of hepatic Gomafu resulted in an increase in random and fasting blood glucose levels in lean mice. In addition, we demonstrated that Gomafu functioned as miR-139 sponge and led to the de-repression of its target gene Foxo1, which played an important role in gluconeogenesis and HGP in hepatocytes. Finally, silenced Foxo1 expression abolished the effect of Gomafu overexpression on gluconeogenesis and glucose production in hepatocytes. Taken together, our data suggested that the increase in Gomafu expression contributed to hepatic IR in obese mice.

## Introduction

The liver plays a critical role in glycolipid metabolism and maintaining blood glucose level within a normal range. Insulin deficiency and glucagon excess in the liver can promote gluconeogenesis, which leads to an increased hepatic glucose production (HGP)^1,2^. Increased HGP is a major contributor to fasting hyperglycaemia in type 2 diabetes mellitus (T2DM)^3^. Therefore, elucidation of the molecular mechanism underlying abnormal activation of gluconeogenesis and HGP is benefit for prevention and treatment of T2DM.

The transcription factor Foxo1 is expressed abundantly in the liver and plays an important role in gluconeogenesis and HGP by regulating expression of key gluconeogenic enzymes such as phosphoenolpyruvate carboxykinase (PEPCK) and glucose-6-phosphatase (G6Pase)^4,5^. The expression and activity of Foxo1 are significantly increased in the livers of patients with diabetes and diabetic mice^6,7^. Liver-specific deletion of Foxo1 in insulin-resistant (IR) mice significantly improved insulin sensitivity and glucose tolerance^8^. Therefore, inhibition of Foxo1 expression and activity in the liver could be a potential strategy for prevention and treatment of IR^9^.

Recent studies have shown that microRNAs are involved in regulating hepatic Foxo1 expression and activity. Our previous study demonstrated that miR-9 was a regulator in hepatic gluconeogenesis and HPG by directly targeting Foxo1 both in vitro and in vivo^10^. Luo et al. confirmed that miR-21 regulated hepatic gluconeogenesis and insulin sensitivity through Foxo1 and its down signaling pathways^11^. MiR-139 had been found to target Foxo1 directly and reduced Foxo1 expression in the liver^12^. However, the role of miR-139 in regulating hepatic gluconeogenesis and IR remains unclear.

Long non-coding RNA Gomafu (also referred to as RNCR2/MIAT) is conserved among mammalian species and mainly located in the nucleus^13^. In recent years, Gomafu has been confirmed to be involved in diabetes-related diseases by competitively sponging microRNA. In diabetic retinopathy mice, Gomafu could upregulate Sp1 expression by sponging miR-29b, ultimately inducing Müller cells apoptosis^14^. Besides, Gomafu could upregulate DAPK2 expression by sponging miR-22−3p, which consequently led to cardiomyocyte apoptosis in a rat model of diabetic cardiomyopathy^15^. Furthermore, Gomafu functioned as a competing endogenous RNA to regulate VEGF levels by sponging miR-150-5p in diabetic retinas and endothelial cells^16^. However, the effect of Gomafu on regulating gluconeogenesis and hepatic IR has not been investigated yet. In this study, we found that Gomafu was significantly upregulated in the liver of ob/ob mice and high-fat diet mice. We also found knockdown of Gomafu decreased HGP and increased insulin sensitivity in the obese mice. We therefore hypothesized that Gomafu might be involved in the regulation of gluconeogenesis and hepatic IR. We tested this hypothesis using AML-12 cells, primary hepatocytes, ob/ob mice, and high-fat diet mice.

## Results

### Hepatic Gomafu expression was decreased in obese mice and in normal mice under fasting conditions

**Fig. 1:**
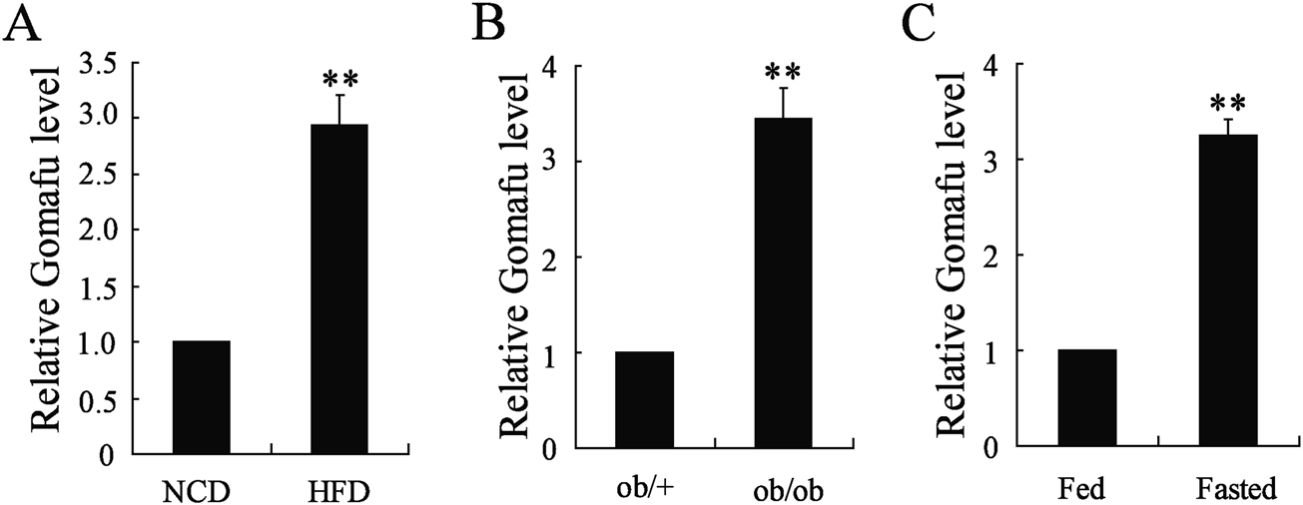
Hepatic Gomafu expression was decreased in obese mice and in normal mice under fasting conditions. **a** Gomafu expression was determined in livers of lean mice fed a normal chow diet (NCD) and obese mice fed a high-fat diet (HFD) (*n* = 3 each group). **b** Gomafu expression was determined in livers of ob/+ and ob/ob mice (*n* = 3 each group). **c** Gomafu expression was determined in livers of fasted C57BL/6J normal mice (mice were fasted for 16 h) and fed C57BL/6J normal mice (*n* = 3 each group). **P < 0.01, compared to control mice or ob/+mice

Previous study showed that Gomafu expression was significantly upregulated in Müller cells in diabetic retinopathy mice^14^. Here we measured the relative expression of Gomafu in two different obese mouse models. We first found that Gomafu expression was obviously increased in livers from obese mice fed a high-fat diet (HFD-fed mice) when compared with lean mice fed the normal chow diet (NCD-fed mice) (Fig. 1A). Next, we observed a significant increase in Gomafu expression in livers from ob/ob obese mice when compared with expression in WT mouse livers (Fig. 1B). In addition, we measured hepatic Gomafu level in C57BL/6J normal mice, following a 16 h fast. Interestingly, fasting increased the Gomafu expression (Fig. 1C).

### NF-κB governed Gomafu expression in vivo and in vitro

**Fig. 2:**
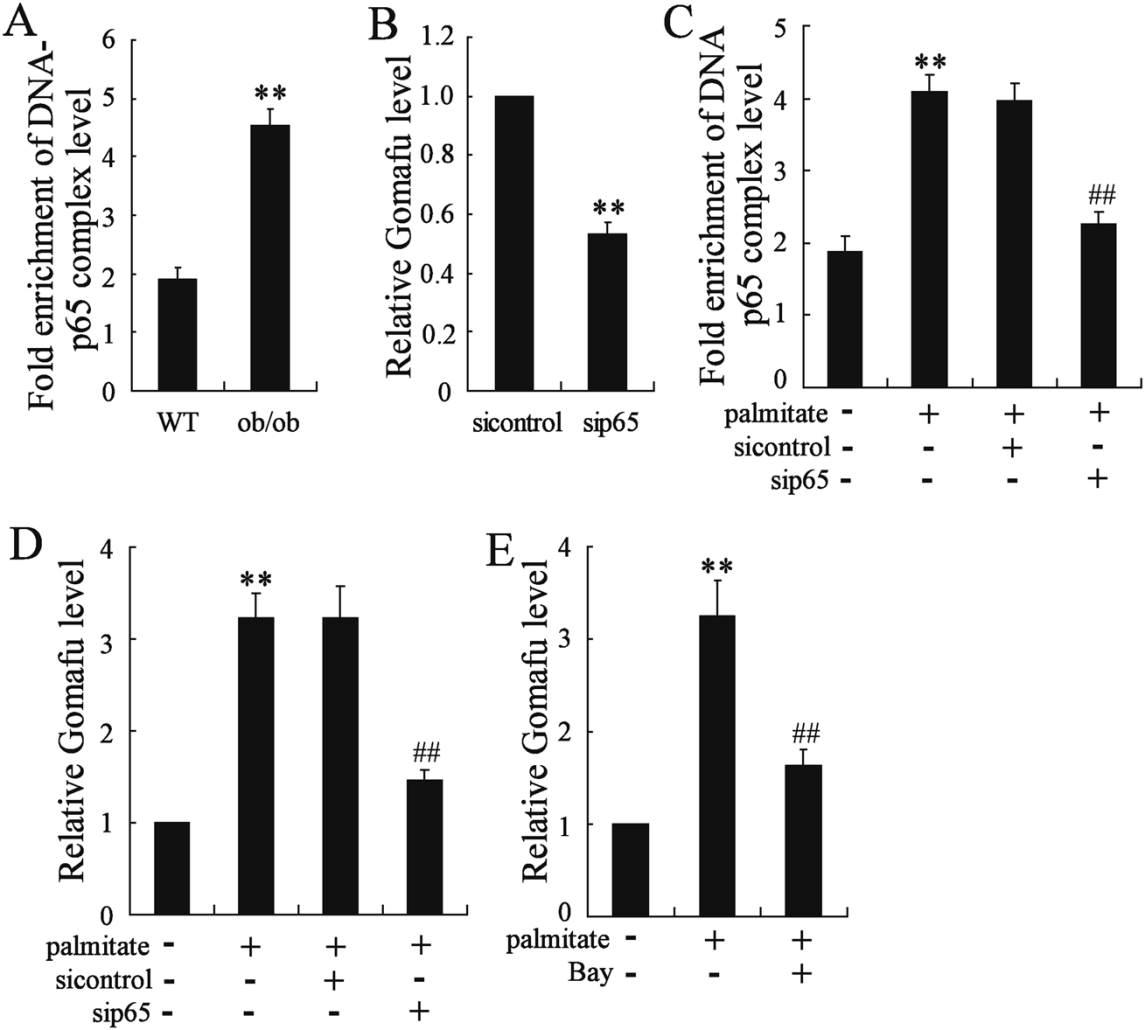
NF-κB governed Gomafu expression in vivo and in vitro. **a** ChIP-qPCR analysis of the capacity for p65 binding to the *Gomafu* promoter was performed in hepatocytes from ob/+ and ob/ob mice. Values are means ± s.d. and were representative of three individual experiments. **b** The ob/ob mice were injected with si-control and sip65 daily for 30 days via the tail vein and Gomafu expression in the liver was determined (*n* = 7 for each group). Primary mouse hepatocytes were transfected with si-control and sip65 for 24 h and then treated with palmitate (0.4 mmol/l) for another 24 h. **c** The capacity for p65 binding to the *Gomafu* promoter and (**D**) Gomafu expression were measured. **e** Primary mouse hepatocytes were treated Bay11-7082 (5 μmol/l) for 2 h, then palmitate (0.4 mmol/l) were added for 24 h. Gomafu expression were measured. Values are means ± s.d. and are representative of three individual experiments. **P < 0.01, compared to control. ^##^*P* < 0.01, compared to palmitate + siNC or palmitate + Bay

NF-κB has been found to regulate Gomafu expression by selectively binding to Gomafu promoter^14^. Quantitative ChIP (qChIP) assay demonstrated the association of p65 with the *Gomafu* promoter region. The binding activity of p65 onto the promoters of *Gomafu* was significantly increased in hepatocytes from ob/ob mice when compared to those from lean mice (Fig. 2A). We also determined the effect of p65 deletion on *Gomafu* expression in ob/ob mice. Injection of sip65 resulted in a decrease of Gomafu level in hepatocytes from ob/ob mice (Fig. 2B).

We also measured the potential involvement of p65 in the expression of Gomafu in vitro. Treatment of hepatocytes with palmitate significantly increased the binding activity of p65 to *Gomafu* promoters and Gomafu expression. These effects could be reversed by deletion of p65 (Fig. 2C,D). In addition, pretreatment with Bay11-7082 could abolish an increase of Gomafu expression in palmitate-exposed hepatocytes (Fig. 2E).

### Knockdown of hepatic Gomafu improved insulin sensitivity in obese mice

**Fig. 3:**
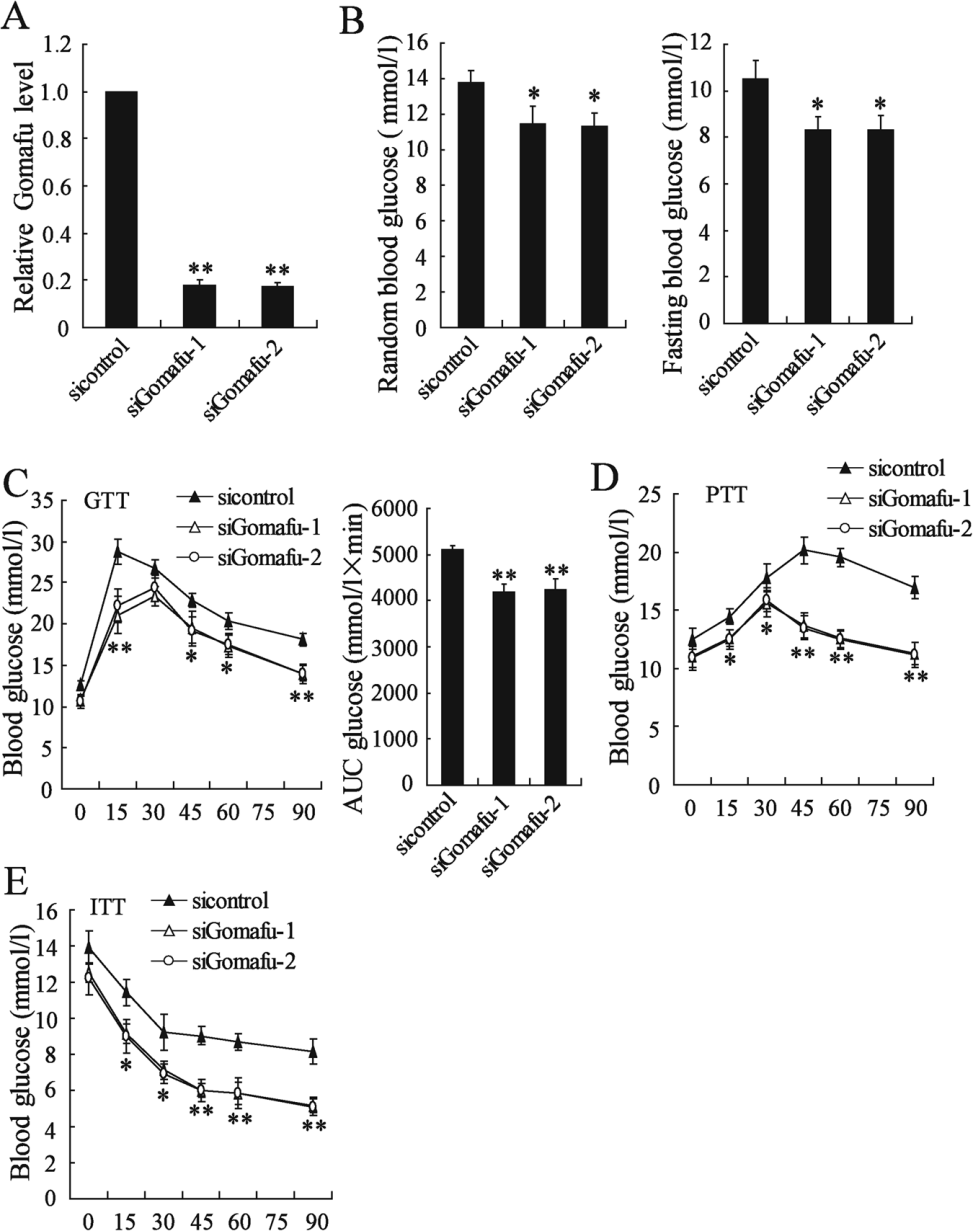
Knockdown of hepatic Gomafu improved insulin sensitivity in obese mice. The ob/ob mice were injected with si-control, siGomafu-1 or siGomafu-2 daily for 30 days via the tail vein. **a** Hepatic Gomafu expression was measured (*n* = 3 each group). **b** Random and fasting blood glucose levels were measured (*n* = 7 each group). **c** GTT was performed in mice fasted for 16 h (*n* = 7 each group) and the area under the curve (AUC) for blood glucose was calculated. **d** PTT was performed in mice fasted for 16 h (*n* = 7 each group). **e** ITT was performed in mice fasted for 4 h (*n* = 7 each group). *P < 0.05, **P < 0.01, compared to si-control group

We next investigated whether the decrease in Gomafu expression in obese mouse livers has any effect on glucose homeostasis and insulin sensitivity. A significant decrease of Gomafu in the hepatocypes from ob/ob mice injected with siGomafu (Fig. 3A). In ob/ob mice and the HFD-fed mice, knockdown of hepatic Gomafu resulted in a decrease the random and fasting blood glucose levels (Fig. 3B and Supplemental Figure-1A). The GTT results showed that decreased hepatic Gomafu expression improved glucose tolerance in ob/ob mice and the HFD-fed mice (Fig. 3C and supplemental Figure-1B). Moreover, The PTT results showed that knockdown of hepatic Gomafu significantly reduced the conversion of pyruvate to glucose in ob/ob mice and the HFD-fed mice, indicating a decrease in gluconeogenesis (Fig. 3D and supplemental Figure-1C). The ITT results also showed that insulin was more efficient in reducing blood glucose level in Gomafu-downregulating ob/ob mice or the HFD-fed mice than in control mice, indicating depletion of hepatic Gomafu improved insulin sensitivity (Fig. 3E and Supplemental Figure-1D). These results indicated that Gomafu functioned as an *in vivo* regulator of glucose homeostasis and insulin sensitivity.

### Overexpression of hepatic Gomafu led to an increase of hepatic glucose production fasting blood glucose levels in lean mice

**Fig. 4:**
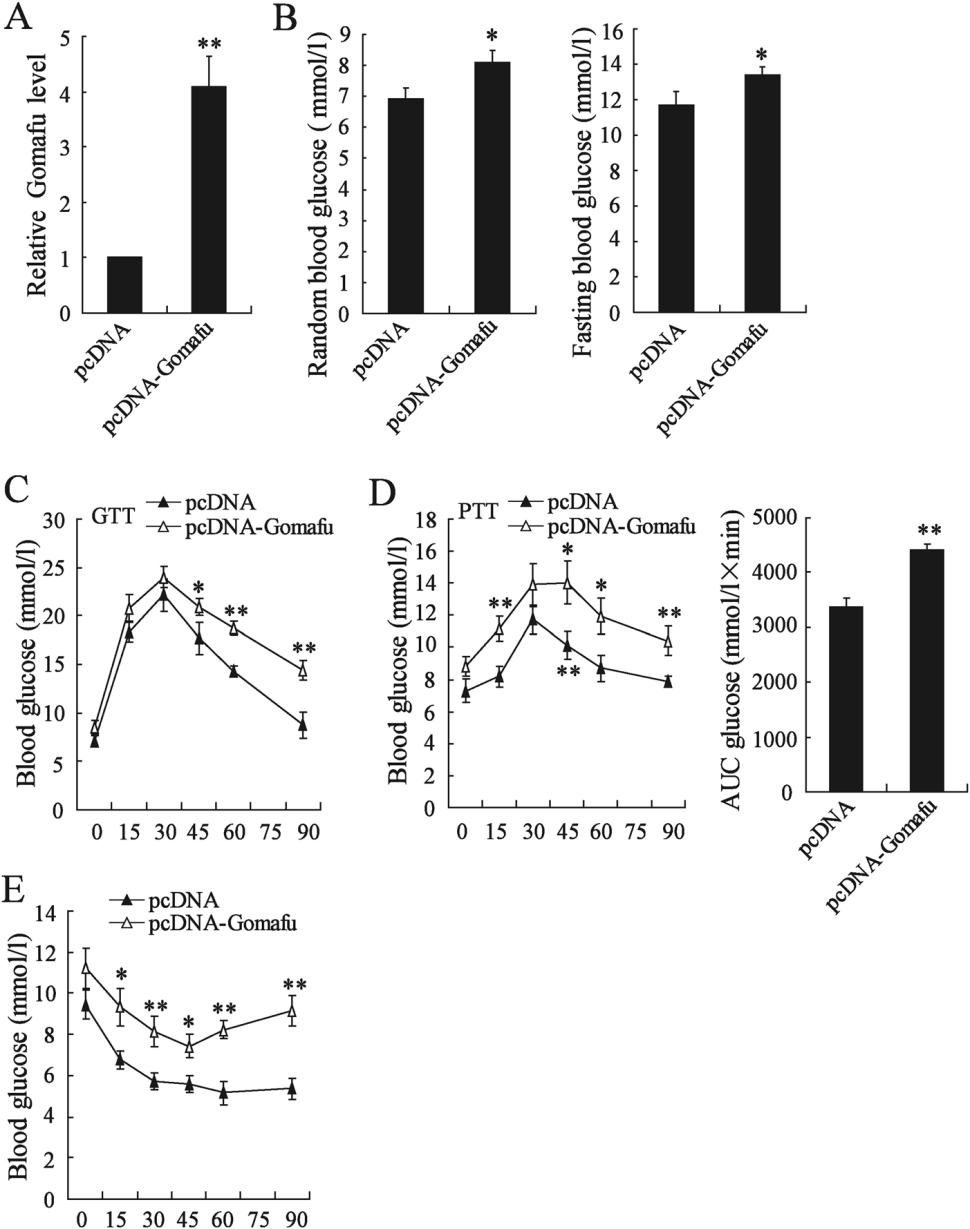
Overexpression of hepatic Gomafu led to an increase of hepatic glucose production fasting blood glucose levels in lean mice. C57BL/6J male mice (8-week-old) were injected with pcDNA or pcDNA-Gomafu daily for 30 days via the tail vein. **a** Gomafu expression was determined in hepatocytes from C57BL/6J mice (*n* = 3 each group). **b** Random and fasting blood glucose levels were determined (*n* = 7 each group). **c** GTT was performed in mice fasted for 16 h (*n* = 7 each group). **d** PTT was performed in mice fasted for 16 h (*n* = 7 each group). **e** ITT was performed in mice fasted for 4 h (*n* = 7 each group). *P < 0.05, **P < 0.01, compared to pcDNA group

We further investigated whether hepatic Gomafu had the effect on insulin sensitivity by injecting pcDNA-Gomafu into C57BL/6J mice via the tail vein^17,18^. The result showed that pcDNA-Gomafu significantly elevated Gomafu expression in hepatocytes (Fig. 4A). Overexpression of hepatic Gomafu also increased random and fasting blood glucose levels (Fig. 4B). The GTT data further showed impairment of glucose tolerance in response to increased hepatic Gomafu expression in lean mice (Fig. 4C). The PTT results showed that the conversion of pyruvate to glucose was significantly increased by upregulation of hepatic Gomafu in lean mice (Fig. 4D). The ITT results showed that insulin was less efficient in reducing blood glucose level in Gomafu-upregulating lean mice (Fig. 4E).

### Gomafu functioned as miR-139 sponge in hepatocytes

**Fig. 5:**
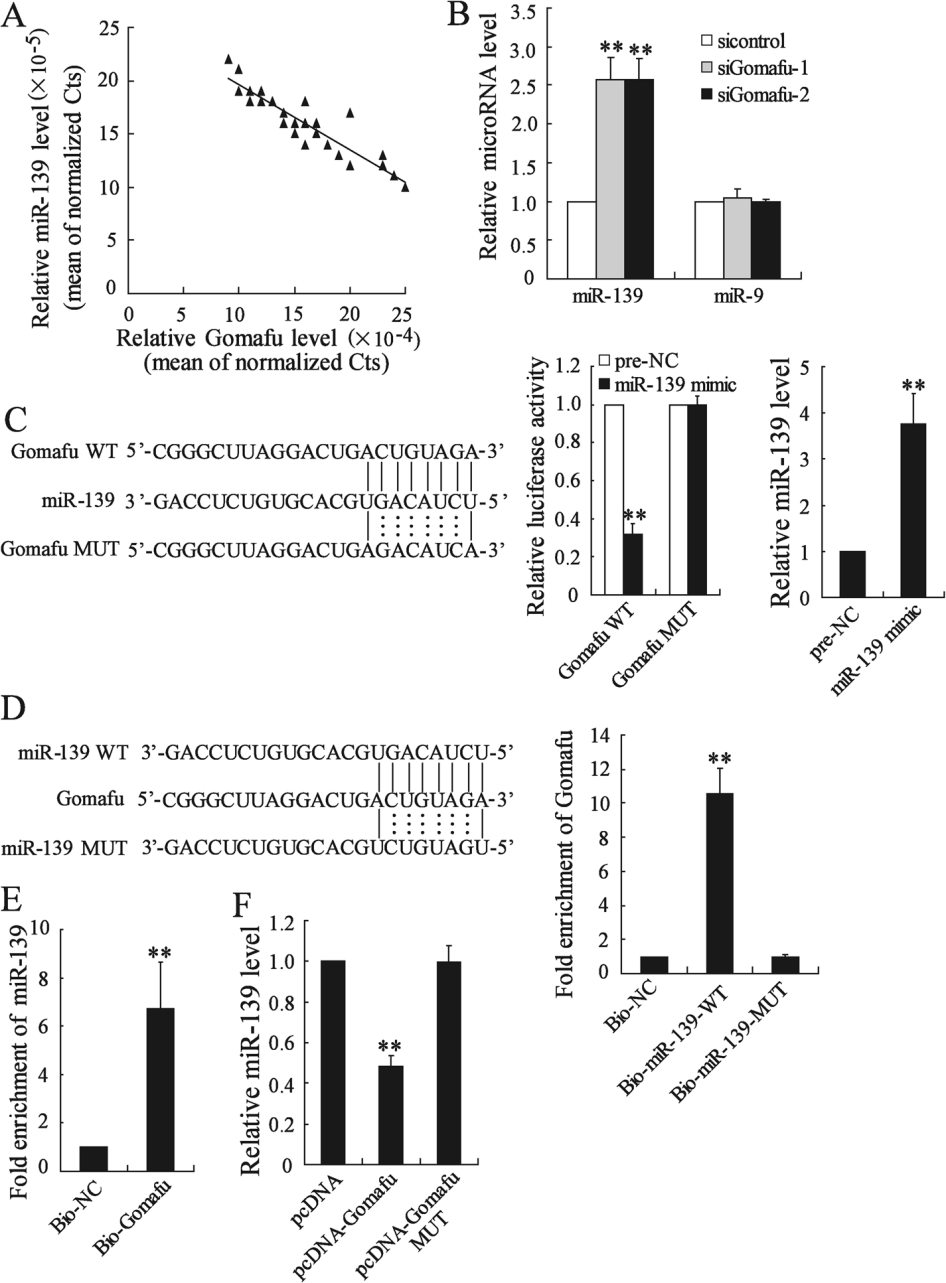
Gomafu functioned as miR-139 sponge in hepatocytes. **a** The correlation between Gomafu and miR-139 expression in 25 hepatic tissues from ob/ob mice. **b** The ob/ob mice were injected with si-control, siGomafu-1 or siGomafu-2 daily for 30 days via the tail vein, miR-139 and miR-9 expression was measured. **c** Sequence alignment of miR-139 with the putative binding sites within WT and the mutated regions of Gomafu. AML-12 cells were co-transfected with miR-139 mimic and Gomafu-WT vector or Gomafu-MUT vector for 48 h, the luciferase activity was measured. The level of miR-139 was measured. **d** WT and the mutated forms of miR-139 sequence were shown. Level of Gomafu in the sample pulled down by biotinylated miR-139 was measured using real-time PCR. **e** Level of miR-139 in the sample pulled down by biotinylated Gomafu probe was measured using real-time PCR. **f** AML-12 cells were transfected with pcDNA-Gomafu or pcDNA-Gomafu MUT for 48 h, the expression of miR-139 was measured. **P < 0.01, compared to si-control, pre-NC, Bio-NC or pcDNA

Gomafu has been demonstrated to function as the endogenous sponge RNA to absorb miRNAs in diabetes-related diseases^14–16^. Our study showed that a significantly negative correlation between Gomafu and miR-139 expression in 25 hepatic tissues from ob/ob mice (*r* = −0.91, *P < *0.001; Fig. 5A). Besides, deletion of Gomafu significantly increased miR-139 expression in livers from ob/ob mice. In contrast to miR-139, Gomafu knockdown had no effect on miR-9 expression (Fig. 5B). Furthermore, we isolated cytoplasmic and nuclear RNA and measured Gomafu expression. The results showed that Gomafu was located in both the nuclear and cytoplasmic fractions of primary mouse hepatocytes (Supplemental Figure-2A). Gomafu siRNA or expression vector modulates nuclear and cytoplasmic Gomafu level (Supplemental Figure-2B). Luciferase reporter assay revealed that upregulation of miR-139 could decrease Gomafu-WT activity, but it had no effect on Gomafu-MUT. The result also showed that miR-139 mimic significantly elevated miR-139 expression in hepatocytes (Fig. 5C). In addition, Gomafu could be pulled down by miR-139, while the introduction of mutations which disrupted the predicted recognition sites between Gomafu and miR-139 resulted in the inability of miR-139 to pull down Gomafu (Fig. 5D,E). The expression of miR-139 was significantly decreased by pcDNA-Gomafu but not by the mutated form of Gomafu in hepatocytes (Fig. 5F). miR-139 had no effect on the expression level of Gomafu (supplemental Figure-3). These results suggested that Gomafu functioned as miR-139 sponge and negatively regulated its expression in hepatocytes.

### Gomafu regulated the expression of miR-139 target gene, Foxo1

**Fig. 6:**
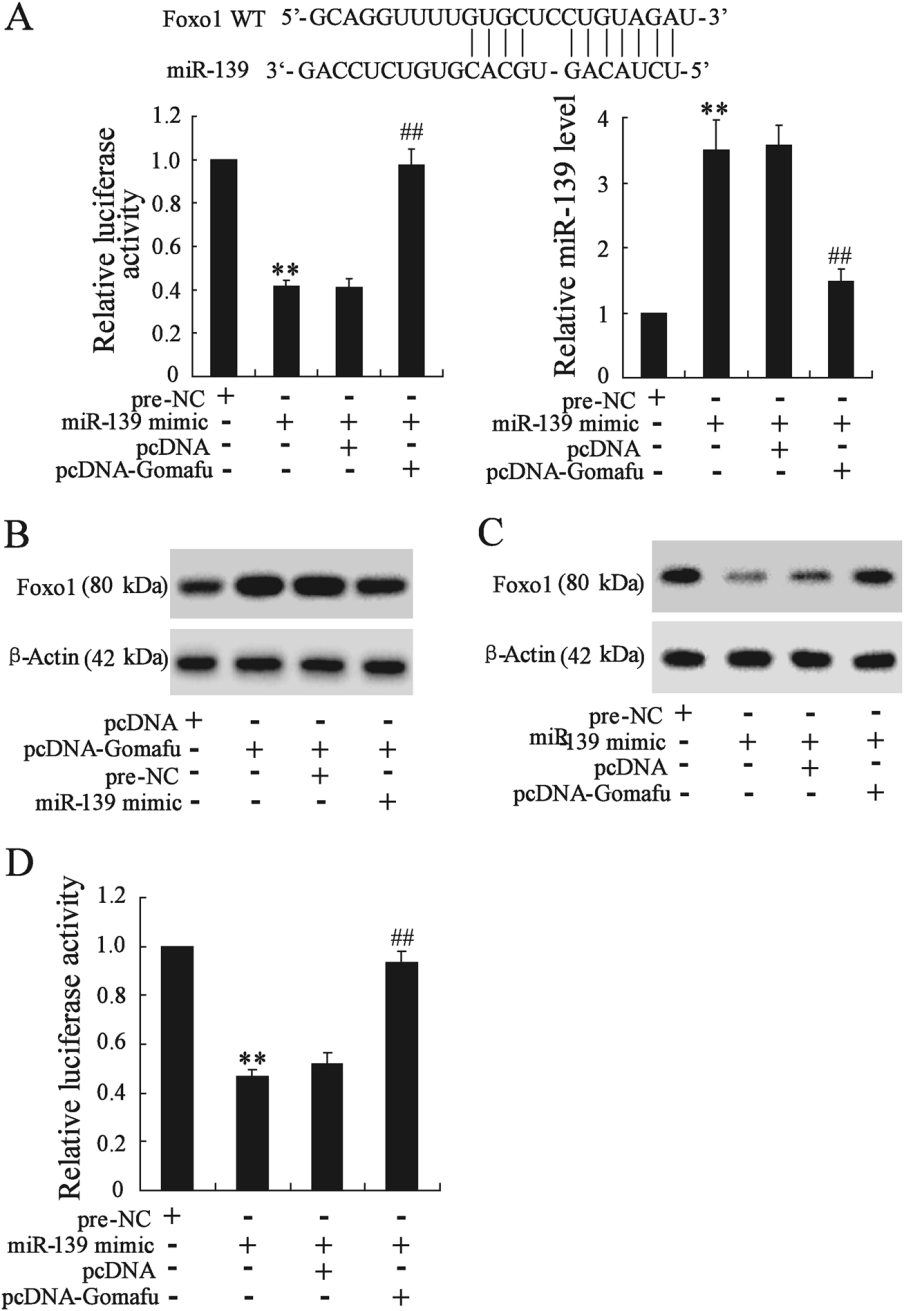
Gomafu regulated the expression of Foxo1. **a** The 3′-UTR of Foxo1 was fused to the luciferase coding region (Luc-Foxo1-WT). Luc-Foxo1-WT and miR-139 mimic was co-transfected into AML-12 cells with pcDNA or pcDNA-Gomafu for 48 h. Luciferase activity was measured using the dual luciferase assay. The level of miR-139 was measured. **b** AML-12 cells were transfected with pcDNA-Gomafu with or without miR-139 mimic for 48 h, Foxo1 expression was measured. **c** AML-12 cells were transfected with miR-139 mimic with or without pcDNA-Gomafu for 48 h, Foxo1 expression was measured. **d** The firefly luciferase reporter construct pGL3-FKHR (containing three FKHR-binding sites) and miR-139 mimic was co-transfected into AML-12 cells with pcDNA or pcDNA-Gomafu for 48 h. Luciferase activity was detected using the dual luciferase assay. **P < 0.01, compared to pre-NC. ^##^*P* < 0.01, compared to miR-139 mimic + pcDNA

It has been demonstrated that miR-139 can target Foxo1 directly and inhibit Foxo1 expression in the liver^12^. To explore whether Gomafu competitively suppressed the binding of miR-139 to Foxo1, we performed luciferase assays in AML-12 cells. The 3′-UTR of Foxo1 was fused to the luciferase coding region (Luc-Foxo1-WT) and transfected into AML-12 cells with miR-139 mimic or pcDNA-Gomafu. The results showed that miR-139 mimic significantly decreased the activity of Luc-Foxo1-WT, whereas Gomafu overexpression abolished miR-139-mediated repression on Luc-Foxo1-WT activity. We also found that miR-139 mimic significantly increased miR-139 expression, which could be reversed by Gomafu overexpression (Fig. 6A). Besides, Gomafu overexpression significantly upregulated Foxo1 level, whereas the increase was reversed when miR-139 level was increased (Fig. 6B). Furthermore, miR-139 overexpression obviously reduced Foxo1 level, and was increased when Gomafu level was increased (Fig. 6C). In addition, we explored the effect of miR-139 and Gomafu on Foxo1 transcriptional activity. The firefly luciferase reporter construct pGL3-FKHR (containing three FKHR-binding sites, a kind gift from Professor Dongming Su of Nanjing Medical University) was transfected into AML-12 cells with miR-139 mimic or pcDNA-Gomafu. The results showed that miR-139 mimic significantly decreased the activity of pGL3-FKHR, whereas Gomafu overexpression abolished miR-139-mediated repression on pGL3-FKHR activity (Fig. 6D). These results suggested that Gomafu might function as a competing endogenous RNA to regulate Foxo1 expression and transcriptional activity by sponging miR-139.

### Gomafu increased gluconeogenesis and HPG by regulating Foxo1

**Fig. 7:**
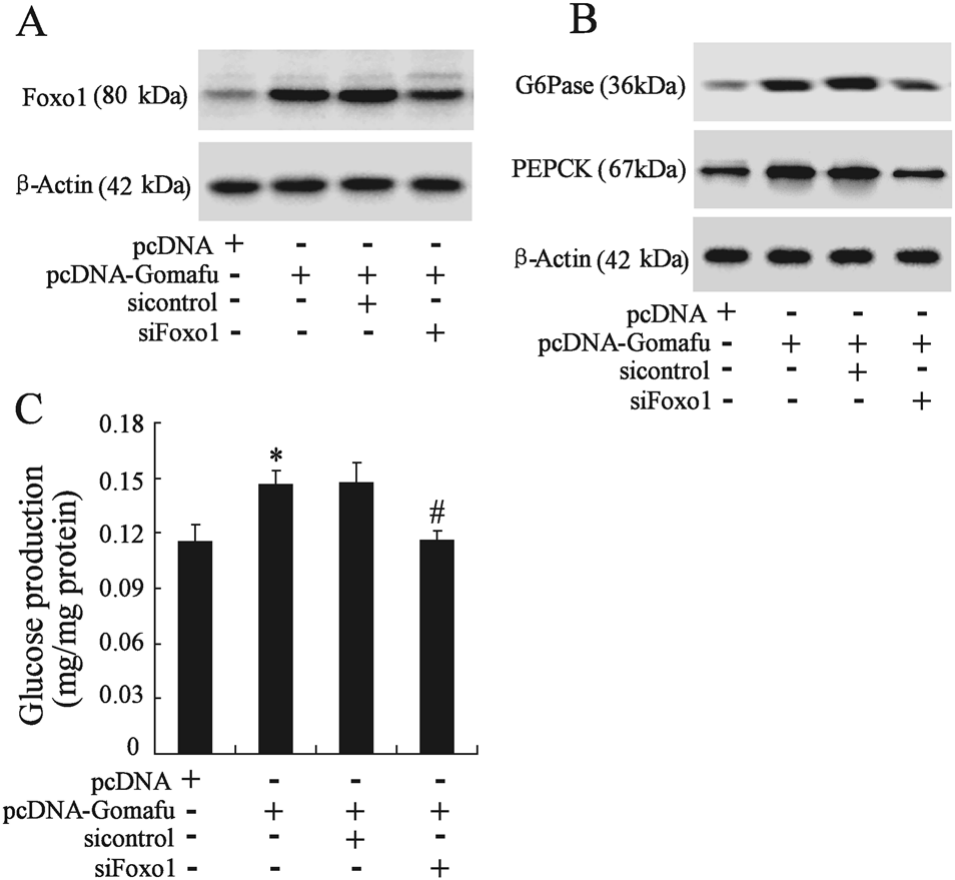
Gomafu increased gluconeogenesis and HPG by regulating Foxo1. Primary mouse hepatocytes were transfected with pcDNA-Gomafu and/or si-Foxo1 for 48 h. The expression of Foxo1 (**a**), PEPCK, and G6Pase (**b**), as well as glucose production in hepatocytes (**c**) was measured. *P < 0.05, compared to pcDNA. #P < 0.05, compared to pcDNA-Gomafu + si-control

The transcription factor Foxo1 was a key regulator of gluconeogenesis and was the target of miR-139 in hepatocytes^4,5^. We explored whether Foxo1 was required for Gomafu overexpression on gluconeogenesis and glucose output. Mouse primary hepatocytes were transfected with pcDNA-Gomafu and siFoxo1. Figure 7A showed a significant increase of Foxo1 expression in hepatocytes transfected with pcDNA-Gomafu; this effect was rescued by transfection with siFoxo1. In addition, overexpression of Gomafu increased PEPCK and G6Pase expression, which was reversed by knockdown of Foxo1 (Fig. 7B). Furthermore, Foxo1 knockdown abolished the increase of glucose production in Gomafu-overexpressing hepatocytes (Fig. 7C). These data indicated that Gomafu overexpression stimulated gluconeogenesis and glucose production by regulating Foxo1.

## Discussion

Long non-coding RNAs have been demonstrated to be involved in the development of hepatic IR and diabetes^19,20^. This study firstly revealed that hepatic LncRNA Gomafu was upregulated thereby contributing to elevated gluconeogenesis and IR in obese mice. It was worth to note that there were no significant differences in body weight, plasma ALT and AST levels and mortality between obese mice injected with siGomafu and those injected with si-control (data not shown). Thus, deletion of hepatic Gomafu expression may be a potential pharmacological strategy to treat IR and diabetes.

Gomafu has been confirmed to be involved in diabetes-related diseases, such as diabetic retinopathy and diabetic cardiomyopathy^14–16^. In this study, we found that Gomafu expression was significantly increased in the livers of obese mice. Besides, our finding also revealed that fasting promoted Gomafu expression in the liver of lean mice. Knockdown of hepatic Gomafu resulted in decreased gluconeogenesis and increased insulin sensitivity, subsequently improving glucose intolerance in high-fat diet mice and ob/ob mice. Gomafu overexpression in the livers of lean mice significantly increased blood glucose levels and the conversion of pyruvate to glucose. Collectively, our data indicated that Gomafu is an important regulator of hepatic gluconeogenesis in vivo.

Previous study reported that NF-κB was involved in regulating Gomafu expression in Müller cells^14^. Here we showed that hepatocytes isolated from HFD-fed and ob/ob mice showed an increase in the binding activity of p65 to the promoters of *Gomafu*. More importantly, deletion of p65 reversed the increase in Gomafu expression in hepatocytes from ob/ob mice. In addition, silencing of p65 or NF-κB inhibitor (Bay11-7082) could alleviate the increase in Gomafu expression in palmitate-exposed hepatocytes. Collectively, these findings clearly demonstrated the role for NF-κB in regulating obesity-induced upregulation of hepatic Gomafu expression.

We further studied the mechanism underlying Gomafu regulation of hepatic glucose metabolism. Gomafu can function as competing endogenous RNAs (ceRNAs) to absorb microRNA and in turn affecting its biological functions^14–16^. Our data showed that Gomafu acted as miR-139 sponge and negatively regulated its expression in hepatocytes (Fig. 5). In previous studies, Gomafu has been demonstrated to be a target of miR-150-5p and its expression can be regulated by miR-150-5p^16,21,22^. However, we discovered that miR-139 could physically interact with Gomafu but had no effect on Gomafu expression in hepatocytes. These suggested that miR-139 was not likely to have canonical miRNA repression on gomafu, which were similar with the mode of miR-23b-3p and MALAT1 in gastric cancer cells^23^. Thus, Gomafu competitively sequestered miR-139 and relieved the inhibitory effect of miR-139 on Foxo1, thereby increasing the expression of Foxo1. Given that Foxo1 played an important role in gluconeogenesis and HGP^24,25^, we further presumed Foxo1 mediated Gomafu function in the regulating glucose metabolism in hepatocytes. This hypothesis was demonstrated by the observation that knockdown of Foxo1 could almost completely abolish the increase in the glucose output and expression of gluconeogenic enzymes in Gomafu-overexpressing hepatocytes.

In conclusion, our study revealed that NF-κB contributed to upregulation of Gomafu expression in the obese mouse liver, leading to elevation of Foxo1 expression by sponging miR-139, and finally to inappropriately activation of gluconeogenesis. Inhibition of Gomafu may be useful for the treatment of obesity and type 2 diabetes.

## Materials and methods

### Reagents

DMEM Nutrient Mix/F12, INSULIN-TRANS-SEL-A and fetal bovine serum (FBS) were obtained from GIBCO (Burlington, ON, USA). Trizol and Lipofectamine 2000 transfection reagent were purchased from Invitrogen Life Technologies (Grand Island, NY, USA). Dexamethasone, Forskolin, Dulbecco’s Modified Eagle’s Medium (DMEM), pentobarbital sodium salt, Cycloheximide (CHX) and the glucose assay kit were purchased from Sigma (Saint Louis, USA). Rabbit polyclonal antibodies against Foxo1, PEPCK and G6Pase as well as mouse polyclonal antibodies against β-Actin were obtained from Santa Cruz Biotechnology (Santa Cruz, CA, USA). Rabbit polyclonal antibody against NF-κB p65 and ChIP assay kits were purchased from Millipore (Billerica, MA, USA).

### Cell culture

The mouse hepatocyte cell line AML-12 was purchased from ATCC and cultured in DMEM Nutrient Mix/F12 supplemented with INSULIN-TRANS-SEL-A, 40 ng/mL of dexamethasone, 10% FBS, 100 units/mL of penicillin, and 100 units/mL of streptomycin. Cells were cultured at 37 °C in a humidified atmosphere containing 95% air and 5% CO_2_.

### Primary isolation and culture of hepatocytes

Hepatocytes were isolated and cultured from the livers of male C57BL/BKS mice as described previously^10^. Cell viability of primary hepatocytes was evaluated by the trypan blue exclusion test and was always higher than 70%. Glucose production in primary hepatocyte was measured using a glucose assay kit (Sigma) and normalized against cellular protein content.

### Animal studies

All animal studies were performed following the guideline established by the Research Animal Care Committee of Yangzhou University (Yangzhou, China). Animals were treated humanely, using approved procedures in accordance with the guidelines of the Institutional Animal Care and Use Committee at Yangzhou University, China. Male C57BL/6J mice and 8-week-old to 12-week-old male ob/+ or ob/ob mice were purchased from the Model Animal Research Center of Nanjing University (Nanjing, China). Diet-induced obese mice were fed ad libitum a high-fat-diet (D12492; Research Diets). Mice were housed under 12/12 h light/dark cycles with free access to food and water. Mice were anesthetized with pentobarbital sodium salt (35 mg/kg) and injected through the tail vein with 2.5 mg/kg body weight of lipid nanoparticles-formulated si-control or siGomafu as described previously^26^.

### Small interfering RNA

Small interfering RNA specific for Gomafu (siGomafu), P65 (siP65), Foxo1 (siFoxo1), and control siRNA (si-control) was synthesized (Ribobio, Guangzhou, China) and transfected using Lipofectamine 2000 in hepatocytes. The sequences of siRNA were: siGomafu-1, 5′-CCAGGCUCCUUUAAACCAATT-3′; siGomafu-2, 5′-GCAGUUCUUAGCUCAUAUATT′; siP65, 5′-CAAGATCAATGGC TACACA-3′; siFoxo1: 5′-UGACUUGGAUGGCAUGUUC-3′; si-control, 5′-GGCC UCAGCUGCGCGACGC-3′.

### Vector construction

Mouse full-length Gomafu cDNA was incorporated 5′ *BamH*I and 3′ *Not*I restriction sites to amplify the Gomafu transcript. To make overexpression constructs, PCR products were digested with *BamH*I and *Not*I (New England Biolabs), purified, and subcloned into the RNA pol-II driven pCDNA3.1 (+) vector immediately after the CMV promoter and upstream of the Bgh polyadenylation site (Invitrogen). The plasmid of pcDNA-Gomafu was sequenced and confirmed to be correct.

### Cytosolic/nuclear fractionation isolation and real-time PCR assay

We isolated cytoplasmic and nuclear RNA using the Cytoplasmic & Nuclear RNA Purification Kit (Norgen, Belmont, CA, USA). Gomafu expressions in cytoplasmic and nuclear fractions were detected by real-time PCR assay. β-Actin and U6 were used as cytoplasmic and nuclear controls, respectively. The sequences of primers were as follow: Gomafu, 5′-TGGAACAAGTCACGCTCGATT-3′ (forward) and 5′-GGTATCCCAAGGAATGAAGTCTGT-3′ (reverse); Foxo1, 5′-TCGTACGCCGACCTCATCA-3′ (forward) and 5′-TCCTTGAAGTAGGGCACGCTC-3′ (reverse); β-Actin, 5′-GCAAGTGCTTCTAGGCGGAC-3′ (forward) and 5′-AAGAAAGGGTGTAAAACGCAGC-3′ (reverse); U6, 5′-CTCGCTTCGGCAGCACA-3′ (forward) and 5′-AACGCTTCACGAATTTGCGT-3′ (reverse).

### Western blot analysis

AML-12 cells, primary mouse hepatocytes and liver tissue from mice were lysed with ice-cold lysis buffer containing: 50 mmol/l Tris–HCl, pH 7.4; 1% NP-40; 150 mmol/L NaCl; 1 mmol/L EDTA; 1 mmol/L phenylmethylsulfonyl fluoride; and complete proteinase inhibitor mixture (one tablet per 10 mL; Roche Molecular Biochemicals, Indianapolis, IN, USA). Individual immunoblots were probed with a rabbit anti-Foxo1, rabbit anti-PEPCK and rabbit anti-G6Pase (all diluted 1:1000) and a mouse anti-β-Actin mAb (diluted 1:5000).

### Quantitative chromatin immunoprecipitation (qChIP) assay

Hepatocytes were lysed in SDS lysis buffer and qChIP assay was performed as previously described^27^. We examined the binding activity of NF-κB p65 on the *Gomafu* promoter using qChIP assay. The sequences of primers used for qChIP were: forward, 5′-GGAGGCTCAACGCGAGAACC-3′ and reverse, 5′-TAGAGCGGAGAAACAGAGCA-3′. The fold difference between p65 antibody-immunoprecipitated samples and those immunoprecipitated with IgG was calculated using 2^−ΔΔCt^.

### Pulldown assay with biotinylated lncRNA-Gomafu DNA probe

Gomafu was transcribed from vector pSPT19-Gomafu and biotin-labeled with the Biotin RNA Labeling Mix (Roche Diagnostics, Indianapolis, IN) and SP6 RNA polymerase (Roche), and purified with an RNeasy Mini Kit (Qiagen, Valencia, CA). The biotinylated Gomafu DNA probe was dissolved in binding and washing buffer, and incubated with Dynabeads M-280 Streptavidin (Invitrogen, CA, USA) at room temperature for 10 min to generate probe-coated beads according to the manufacturer’s protocol. Then, AML-12 cell lysates were incubated with the probe-coated beads, and the RNA complexes bound to these beads were eluted and extracted for real-time PCR analysis.

### Pulldown assay with biotinylated miR-139

AML-12 cells were transiently transfected with biotinylated miR-139, miR-139-Mut, and negative control (Ribobio, Guangzhou, China), harvested and lysed 48 h after transfection. Fifty microliter of the samples were aliquoted for input. The remaining lysates were incubated with Dynabeads M-280 Streptavidin (Invitrogen, CA, USA) according to the manufacturer’s protocol. In brief, the washed beads were treated in RNase-free solutions and incubated with equal volume of biotinylated miR-139 for 10 min at room temperature in binding and washing buffer on a rotator. Then, the beads with the immobilized miR-139 fragment were incubated with 10 mM EDTA with 95% formamide at 65 °C for 5 min. The bound RNAs were purified using Trizol for the real-time PCR analysis.

### Luciferase reporter assays

The 3′-UTR of mouse Foxo1 or Gomafu was amplified from mouse genomic DNA and individually inserted into the pmiR-RB-REPORT (Ribobio, Guangzhou, China) using the XhoI and NotI sites. Similarly, the fragment of Foxo1 3′-UTR or Gomafu mutant was inserted into the pmiR-RB-REPORT control vector at the same sites. For reporter assays, AML-12 cells were co-transfected with wild-type (mutant) reporter plasmid and miR-139-Ribo mimic (miR-Ribo negative control). Luciferase activity was measured 48 h post-transfection using the Dual Luciferase Reporter Assay System (Promega, Madison, WI, USA).

### Glucose tolerance test (GTT), insulin tolerance test (ITT), and pyruvate tolerance test (PTT)

GTT was performed by an intraperitoneal injection of d-glucose (2 g/kg body weight, i.p.) after a 16 h overnight fast. Blood glucose levels were measured at 0, 30, 60, and 90 min after injection and the area under the curve (AUC) for blood glucose was calculated. For ITT, mice were fasted for 6 h and then injected with human insulin (Novo-Nordisk, Bagsværd, Denmark) at 0.75 U/kg body weight. Blood glucose levels were measured at 0, 15, 30, 60, and 90 min. PTT was performed to estimate gluconeogenesis. Mice were starved for 16 h and then injected intraperitoneally with pyruvate (2 g/kg body weight, i.p.) dissolved in saline. Blood glucose levels were measured in the tail blood. Blood glucose levels were measured using the hexokinase Method (Thermo Fisher Scientific, Lafayette, CO, USA).

### Statistical analysis

Statistical analyses were performed using statistical analysis software SPSS 19.0. Data were expressed as the mean ± SD. Analysis of variance (ANOVA) was used to determine the statistical differences among the groups. A *P*-value of <0.05 and are provided in the figures. A *P*-value < 0.05 was considered statistically significant.

## Electronic supplementary material

Supplemental Figures
