# Supplementary material for: Long noncoding RNA Gomafu upregulates Foxo1 expression to promote hepatic insulin resistance by sponging miR-139-5p
Source: Cell Death Dis. 2018 Feb 19;9(3):289. doi: 10.1038/s41419-018-0321-7 (PMC5833404; doi:10.1038/s41419-018-0321-7)
Supplement: Supplementary file 1 — Supplemental Figures [file 41419_2018_321_MOESM1_ESM.doc]

**Supplemental Figure-1 Knockdown of hepatic Gomafu improved insulin sensitivity in obese mice.** Mice were fed a high-fat diet (HFD) for 15 weeks, and then were injected with sicontrol, siGomafu-1 or siGomafu-2 daily for 30 days via the tail vein. (A) Random and fasting blood glucose levels were measured (n=7 each group). (B) GTT was performed in mice fasted for 16 h (n=7 each group) and the area under the curve (AUC) for blood glucose was calculated. (C) PTT was performed in mice fasted for 16 h (n=7 each group). (D) ITT was performed in mice fasted for 4 h (n=7 each group). *P < 0.05, **P < 0.01, compared to sicontrol group.


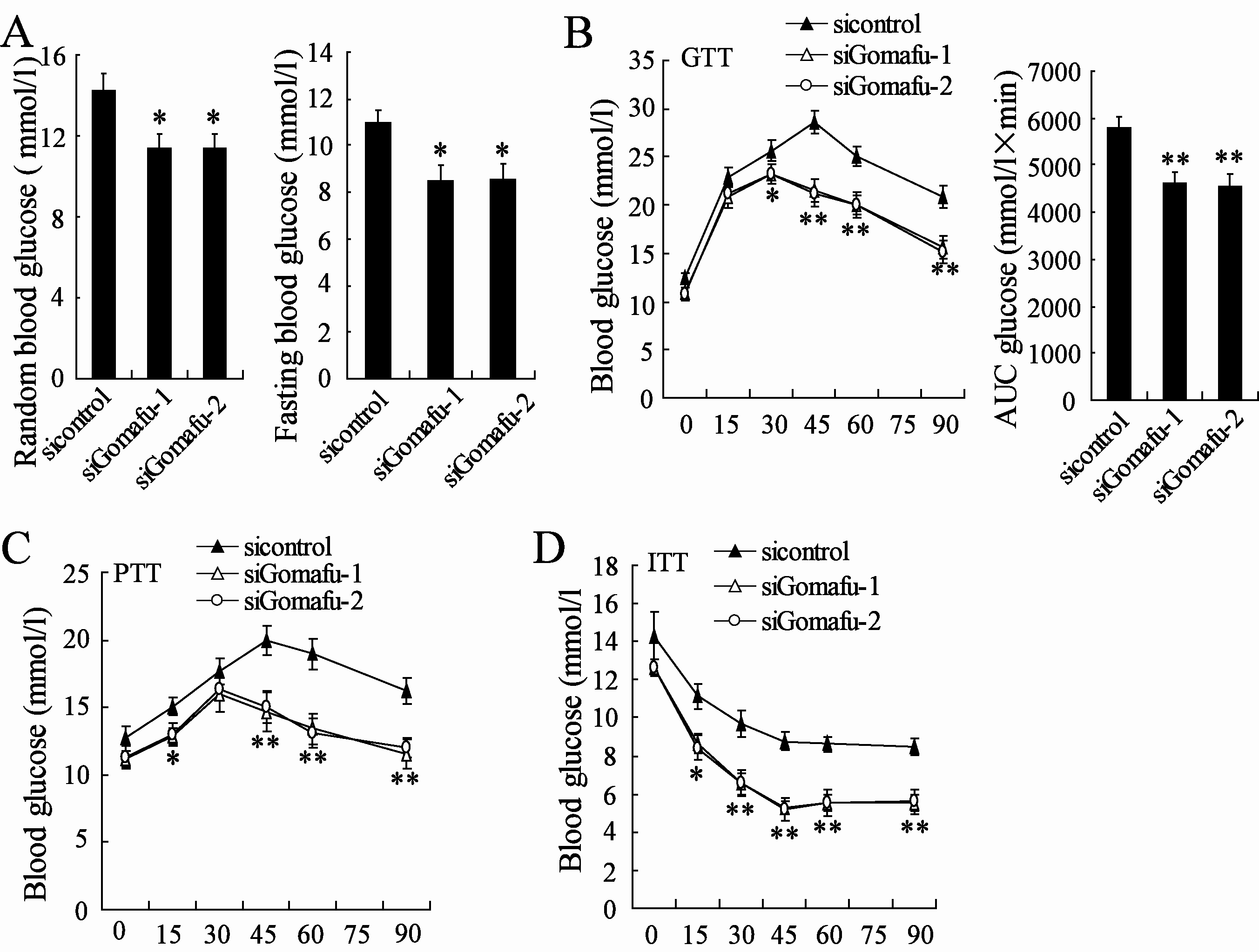


**Supplemental Figure-2 The expression of nuclear/cytoplasmic gomafu in hepatocytes.** cytoplasmic and nuclear RNA was isolated using the Cytoplasmic & Nuclear RNA Purification Kit. (A) Gomafu expression in cytoplasmic and nuclear fractions was detected by RT-PCR in hepatocytes. β-Actin and U6 were used as cytoplasmic and nuclear controls, respectively. (B) Primary mouse hepatocytes were transfected with pcDNA-Gomafu or si-Gomafu for 48 h, the expression of Gomafu was measured by real-time PCR. **P < 0.01, compared to pcDNA or si-control.

**
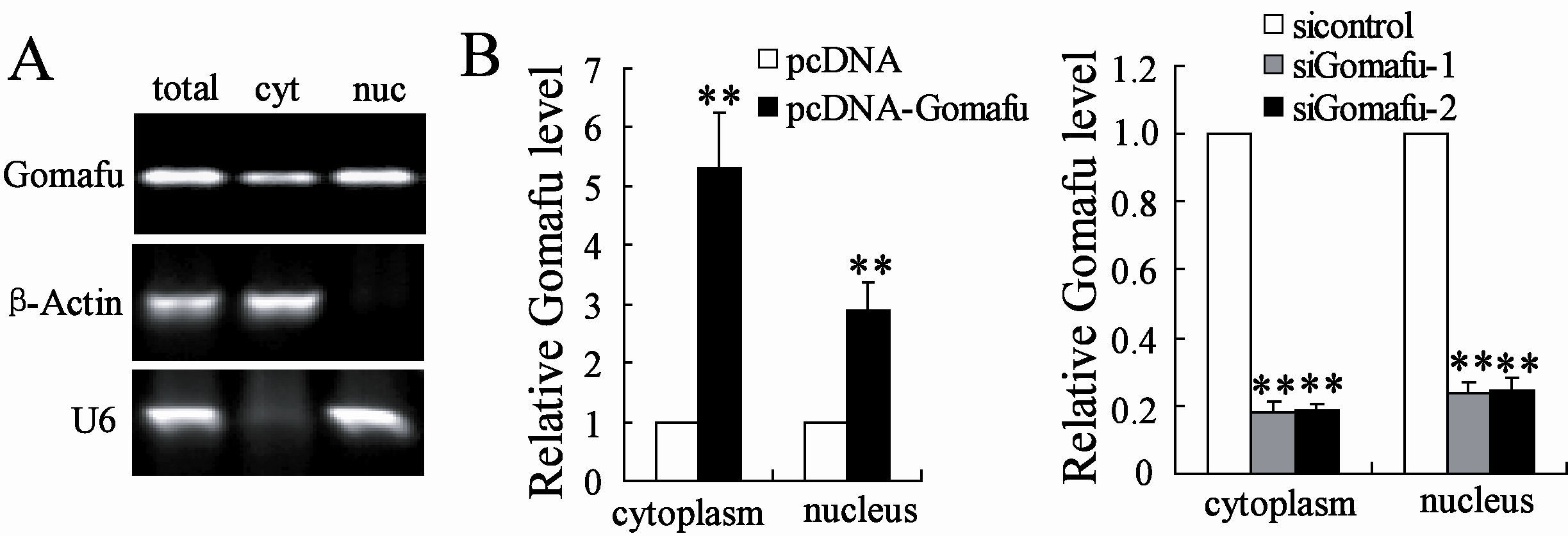
**

**Supplemental Figure-3 miR-139 had no effect on the expression of Gomafu.** Primary mouse hepatocytes were transfected with miR-139 mimic or miR-139 inhibitor for 48 h, the expression of Gomafu was detected by real-time PCR.

**
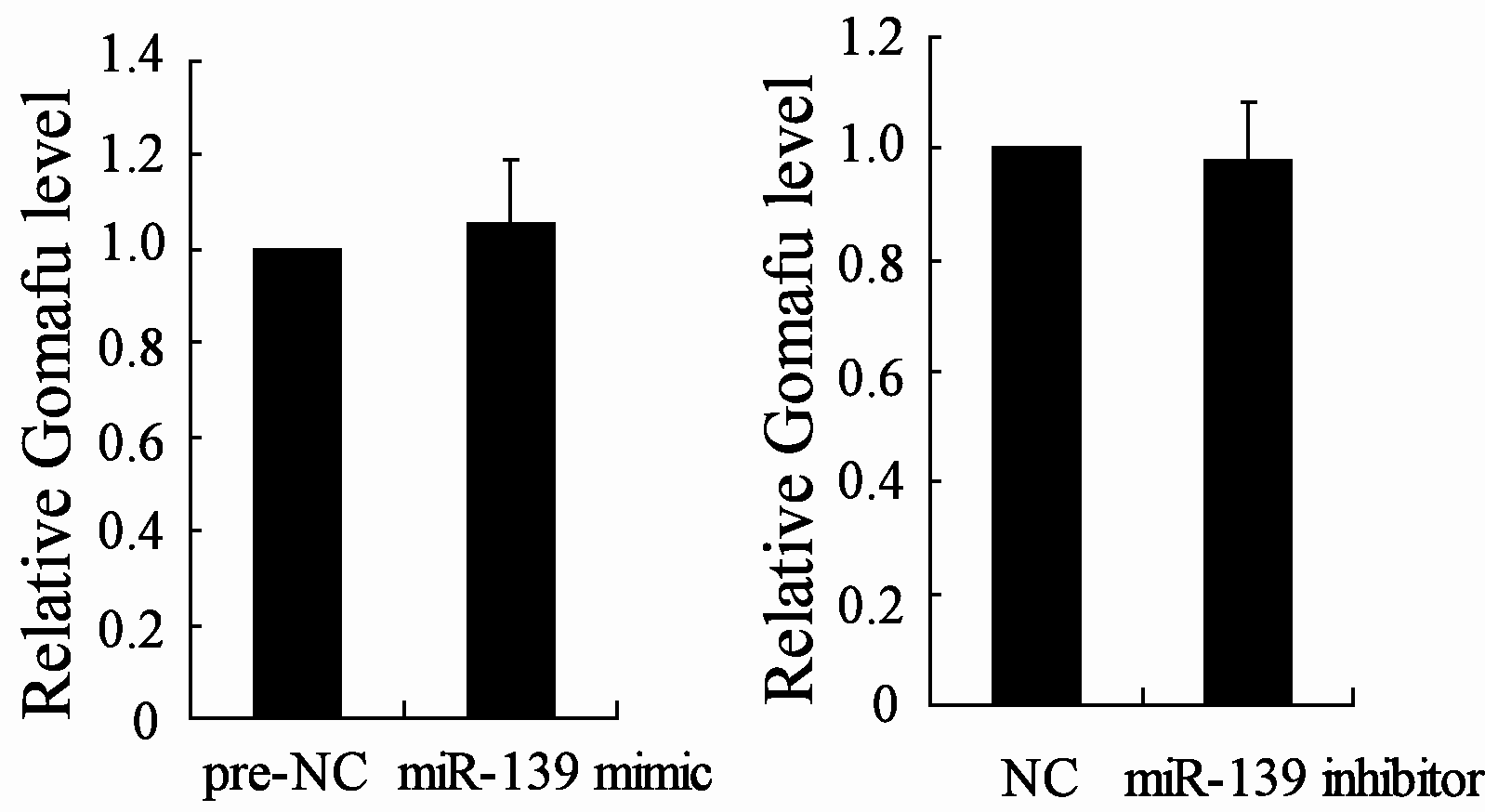
**
